# Draft Genome Sequence of a Novel *Mucilaginibacter* Member Isolated from Brazilian Amazon Soil

**DOI:** 10.1128/genomeA.01033-16

**Published:** 2016-10-06

**Authors:** Sérgio Amorim de Alencar, Flávio Silva Costa, Gisele Regina Rodrigues, Cristine Chaves Barreto

**Affiliations:** Programa de Pós-Graduação em Ciências Genômicas e Biotecnologia, Universidade Católica de Brasília, Brasília-DF, Brazil

## Abstract

Bacteria from the *Mucilaginibacter* genus are still poorly understood, although their importance has been shown by recent reports describing great quantities of biofilms produced in their colonies. We report the draft genome sequence of a novel *Mucilaginibacter* member, comprising 8 contigs, totaling 5,478,589 bp and 4,876 predicted coding sequences.

## GENOME ANNOUNCEMENT

The *Mucilaginibacter* genus belongs to the *Bacteroidetes* phylum and the *Sphingobacteriaceae* family, and was first described in 2007 (1). The members of this genus are facultative aerobic, Gram-negative bacteria, and have been found in aquatic, terrestrial, and acidic habitats (1, 2). Little is known about this genus and their enzymes, but some are biofilm-producing bacteria and have enzymes responsible for the disruption of polysaccharides (1, 3).

In order to evaluate the potential of bacteria from the *Mucilaginibacter* genus in the depolymerization process of polysaccharides, several *Mucilaginibacter* samples were obtained from Amazon soil converted to pasture located in Rondônia State, Brazil (4). The 16S rRNA sequence similarity analysis showed that they are novel members of the *Mucilaginibacter* genus, presenting 86% of similarity with the sequence of *Mucilaginibacter paludis*. One bacterial sample that showed high enzymatic activity was selected for DNA extraction using the MasterPure complete DNA and RNA purification kit (Epicentre Biotechnologies, USA), according to the manufacturer’s protocol. The genomic DNA library was constructed using a Nextera XT library prep kit (Illumina, Inc., San Diego, CA). Library quantity and quality was assessed using the Agilent 2100 Bioanalyzer (Agilent Technologies). Finally, the DNA library was sequenced (2 × 300 bp) on a MiSeq platform (Illumina, San Diego, CA) at the Catholic University of Brasília, Brazil. Then, sequencing data was converted to fastq format using the MiSeq reports program, producing 14,288,200 raw reads. In order to perform quality trimming and adapter removal, pre-processing was carried out with the Trimmomatic tool (5) using the following parameters: sliding window: 4:20; leading: 3; trailing: 3; minlength: 36. Quality assessment of the pre-processed data was carried out using the FastQC tool version 0.11.5 (http://www.bioinformatics.babraham.ac.uk/projects/fastqc), which confirmed that poor quality bases were removed. In order to account for the possibility of the paired-end reads overlapping, FLASH software was used to merge pairs of reads when the original DNA fragments were shorter than twice the length of the reads (6). *De novo* genome assembly was carried out with SPAdes version 3.7.1 (7), resulting in a draft genome size of 5.48 Mbp with eight contigs, *N*_50_ of 9,709,906 bp, and G+C content of 45.8%. The genome was then annotated using the RAST server (8, 9), which identified 4,876 coding sequences and 49 RNA genes, and 370 subsystems, which represent 32% of assigned sequences. The average nucleotide identity (ANI), a similarity index between two genomes (10), was used to compare this assembly to the genomes of *Mucilaginibacter* species available at the NCBI genome database and the Joint Genome Institute. The ANI values obtained for *M. paludis* (76.1%), *M. frigoritolerans* (76.1%), *M. gossypiicola* (76.5%), *M. polytrichastri* (75.7%), *M. gossypii* (77.0%), *M.* sp*.* OK098 (76.1%), *M.* sp*.* YR332, and *M.* sp*.* L294 (75.9%) indicate that this assembly represents in fact a novel species.

### Accession number(s).

This whole-genome shotgun project has been deposited in DDBJ/ENA/GenBank under the accession no. MDJS00000000. The version described in this paper is the first version.
